# DICE, an efficient system for iterative genomic editing in human pluripotent stem cells

**DOI:** 10.1093/nar/gkt1290

**Published:** 2013-12-04

**Authors:** Fangfang Zhu, Matthew Gamboa, Alfonso P. Farruggio, Simon Hippenmeyer, Bosiljka Tasic, Birgitt Schüle, Yanru Chen-Tsai, Michele P. Calos

## Abstract

To reveal the full potential of human pluripotent stem cells, new methods for rapid, site-specific genomic engineering are needed. Here, we describe a system for precise genetic modification of human embryonic stem cells (ESCs) and induced pluripotent stem cells (iPSCs). We identified a novel human locus, *H11*, located in a safe, intergenic, transcriptionally active region of chromosome 22, as the recipient site, to provide robust, ubiquitous expression of inserted genes. Recipient cell lines were established by site-specific placement of a ‘landing pad’ cassette carrying *attP* sites for phiC31 and Bxb1 integrases at the *H11* locus by spontaneous or TALEN-assisted homologous recombination. Dual integrase cassette exchange (DICE) mediated by phiC31 and Bxb1 integrases was used to insert genes of interest flanked by phiC31 and Bxb1 *attB* sites at the *H11* locus, replacing the landing pad. This system provided complete control over content, direction and copy number of inserted genes, with a specificity of 100%. A series of genes, including mCherry and various combinations of the neural transcription factors LMX1a, FOXA2 and OTX2, were inserted in recipient cell lines derived from H9 ESC, as well as iPSC lines derived from a Parkinson’s disease patient and a normal sibling control. The DICE system offers rapid, efficient and precise gene insertion in ESC and iPSC and is particularly well suited for repeated modifications of the same locus.

## INTRODUCTION

Pluripotent stem cells, including embryonic stem cells (ESCs) and induced pluripotent stem cells (iPSCs), offer tremendous potential—for understanding human development and disease mechanisms and for use in drug screening and cell therapy strategies. New methods are needed to insert genes in these cells in a controlled manner, as current technologies have serious limitations. For example, gene insertion mediated by retroviruses, lentiviruses, transposons and non-homologous end-joining (NHEJ) results in random integration. The consequent lack of control over transgene insertion site, copy number and orientation compromises the precision of experiments. Moreover, these methods often have limits on the size of the DNA that can be inserted.

Gene insertion using the canonical phiC31 phage integrase can be performed with high stringency (1–4) and has been used to insert BACS of over 100 kb in size (5). Integration mediated by phiC31 and certain related phage integrases such as Bxb1 provides unidirectional recombination that is tightly restricted to each enzymes’ own small *attB* and *attP* recognition sites (3,6,7). However, these target *att* sites are not naturally present in mammalian genomes. To enable high recombination frequency, the *att* sites must be introduced into the genome. Homologous recombination (HR) provides a route to precise gene addition (8), but spontaneous recombination is inefficient, size-sensitive and requires significant homology arms, thus making it laborious to perform. Recombination frequency could potentially be reduced even further if the cells under study exhibit disease pathology. The frequency of HR can be stimulated by provision of a double-strand break at the target site; the break can be produced by zinc finger nucleases (9), TALENs (10) and CRISPR/Cas9 technologies (11). However, creating double-strand breaks may have undesirable side effects, including cellular toxicity, off-target recombination and sequence alterations near the target site.

**Figure 1.**
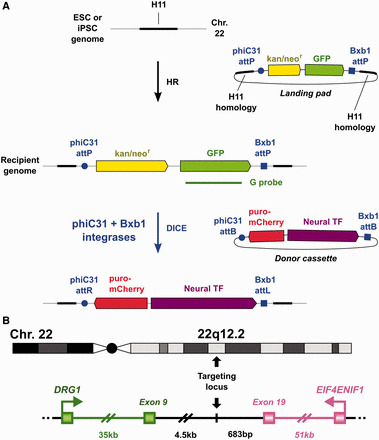
DICE strategy and *H11* location. **(A)** Schematic diagram describing a two-step process for robust and easily repeatable placement of any genes into human pluripotent stem cells. First, the *attP*-flanked Neo-GFP landing pad is introduced into the *H11* locus by HR to generate a recipient cell genome. The position of the probe (G probe) used to characterize the recipient cell lines by Southern blotting is indicated. Then, the donor gene cassette is recombined into this location by DICE. In this case, the donor cassette carries genes for neural transcription factors (F), as well as puromycin resistance and mCherry genes for selection and screening. **(B)** Location of the *H11* locus in the human genome. *H11* resides in an intergenic region on chromosome 22q12.2, flanked by the *DRG1* and *EIF4ENIF1* genes. The distances from *H11* to the terminal exons of the two flanking genes are indicated.

Here, we combine the attractive features of site-specific integrases and HR to create a new method for precise gene addition in human pluripotent stem cells. This method, called dual integrase cassette exchange (DICE), offers complete control over the content, orientation and copy number of gene insertion and is expected to have no size limits. In the DICE method, HR is utilized for placement of a small ‘landing pad’ equipped with *attP* sites for two phage integrases that recognize only their own small recognition sites (Figure 1A). The landing pad was directed to a novel locus that was chosen by bioinformatics analysis and expected to provide robust transcription in all cell types. The murine *Hipp11* locus was first described by Hippenmeyer *et al.* (12) and further validated in mice for integrase-mediated transgenesis (13) and widespread expression (14). The orthologous human *H11* locus is described here for the first time. We pursued both spontaneous and TALEN-assisted HR for placement of the landing pad into *H11*, uncovering a requirement for assisted recombination when modifying a disease iPSC line with significant pathology. Once recipient cell lines were created that bore the landing pad in the favorable *H11* locus, a cassette-exchange strategy was applied to insert genes of interest. The cassette exchange was mediated by two different site-specific integrases, phiC31 and Bxb1, each directing highly accurate recognition and synapsis of its own sites, with no cross-recognition of the *att* sites of the other integrase. In this way, only the desired sequence was inserted, devoid of unneeded flanking plasmid sequences that could be deleterious (13). Furthermore, only a single copy was inserted, as is typical for integrases, whether one or two integrases are used. By using two integrases, the reaction was invariably precise, producing only the desired orientation. This feature eliminated unwanted integration of the plasmid backbone and ensured that the transgene was always inserted in the same orientation with respect to the genome. This outcome permits various transgenes to be compared faithfully in the identical genomic context, eliminating potential variation in expression due to orientation. 

In this study, insertion of GFP and mCherry marker genes confirmed robust gene expression at *H11*. Moreover, by applying the DICE strategy, we were able rapidly to produce a large series of ESC and iPSC lines carrying different combinations of neuronal transcription factor genes. These lines will be useful for analyzing differentiation of stem cells into dopaminergic neurons, as a potential therapeutic approach for Parkinson’s disease (PD) (15). This study demonstrates that DICE is a simple strategy for precise gene addition that is effective in human ESC and iPSC and is particularly useful for creating a series of cell lines starting from the same parent line, with various genes inserted at the same chosen, desirable location.

## MATERIALS AND METHODS

### Plasmid construction

PCR reactions for plasmid construction were performed with either Phusion DNA polymerase (New England Biolabs, Ipswich, MA, USA) or KAPA HiFi HotStart ReadyMix (2×) (KAPA Biosystems, Woburn, MA, USA). All DNA fragments amplified by PCR were completely sequenced after cloning. Primers used to generate the homology arms are listed in Supplementary Table S1. For the landing pad plasmid p2attNG, the structure of the Neo and GFP exchange cassette is provided in Supplementary Figure S1A. The homology arms for HR at the human *H11* locus in the landing pad plasmid were 5 and 3 kb in length, while homology arms of 400 bp on each side were used for TALEN-assisted recombination. For the donor plasmid, the structure of plasmid p2attPC-LFO is provided in Supplementary Figure S1B. The constructs with two of the three transcription factors and the control plasmid lacking transcription factors were generated based on the p2attPC-LFO plasmid. Transcription factor cDNA sequences were purchased from DNASU, the DNA repository at Arizona State University. Plasmid sequences will be made available upon request.

For TALEN targeting plasmid construction, TALEN repeat variable di-residues (RVDs) were designed by the TAL Effector Nucleotide Targeter 2.0 (10,16) (spacer 15–24, RVDs = 15–20). TALEN-encoding plasmids were assembled using the Golden Gate TALEN and TAL Effector Kit 1.0 and its protocol for assembly of the TALEN-encoding plasmid (Addgene; www.addgene.org). The mammalian expression vector with modified FokI was a kind gift from Dr Matthew Porteus, Stanford University. The efficiency of induction of double-strand breaks by transcription activator-like effector (TALE) pairs was determined by using the SURVEYOR® Mutation Detection Kit (Transgenomic Inc., Omaha, NE, USA).

### Cell culture and differentiation

Human pluripotent stem cells, including H9 ESC, PI-1761 and PI-1754 iPSC, and subclones derived from these cells, were cultured on irradiation-inactivated mouse embryo fibroblasts (MEFs) in human stem cell culture medium consisting of Dulbecco’s modified eagle medium/F12 (DMEM/F12), 20% knockout serum replacement, 0.1 mM non-essential amino acids (NEAA), 2 mM glutamax, 0.1 mM β-mercaptoethanol (2-ME) (all from Invitrogen, Carlsbad, CA, USA) and 10 ng/ml bFGF (R&D Systems, Minneapolis, MN, USA). Culture medium was changed daily. H9 and PI-1761 cells were passaged every 5–7 days using 1 mg/ml collagenase IV (Invitrogen), while PI-1754 PD patient iPSCs were manually passaged every 7–10 days.

For embryoid body (EB)-mediated differentiation, cells were incubated with 1 mg/ml dispase (Invitrogen) for 15 min, washed, and then colonies were harvested and transferred from 6-well plates to ultra low attachment surface 6-well plates (Corning, Tewksbury, MA, USA) in differentiation medium containing DMEM/F12, 15% fetal bovine serum (Invitrogen), 0.1 mM NEAA, 2 mM glutamax and 0.1 mM 2-ME. Medium was changed every 2–3 days. After 8 days in suspension culture, EBs were collected, reseeded onto 24-well plates pre-coated with 0.1% gelatin (Sigma Aldrich, St. Louis, MO, USA) and cultured for another 8 days in the same medium.

### Nucleofection and stable line generation

Nucleofection was performed using the P3 Primary Cell 4D-Nucleofector® X Kit L (Lonza, Walkersville, MD, USA) according to the manufacturer’s instructions. Briefly, cells were dissociated with Accutase (Millipore, Billerica, MA, USA) into single cells, depleted of MEF feeders and counted. Then, 0.8–1.6 × 10^6^ cells were nucleofected in one cuvette and quickly reseeded onto multi-antibiotic resistant DR4 MEF cells (Applied StemCell, Menlo Park, CA, USA) freshly prepared 1 day earlier. The Rho kinase inhibitor Y27632 (Tocris Bioscience, Bristol, UK) was used 24 h before and after nucleofection to promote cell survival.

For generation of recipient ESC and iPSC lines, 5 μg landing pad plasmid was used for spontaneous HR, and 8 μg landing pad plasmid and 1 μg TALEN-encoding plasmids were used for TALEN-mediated recombination. For DICE, 4 μg was used for each of phiC31 integrase [pCS-kI; (17)], Bxb1 integrase [pCMV-Bx; (6)] and *attB* donor plasmids. Cells were selected with either 50 μg/ml G418 (Invitrogen) for HR or 500 ng/ml puromycin (Invitrogen) for DICE, starting 2–4 days after nucleofection and exposing cells to puromycin for only 2–3 days. Surviving clones that were mCherry-positive and GFP-negative were picked 2 weeks after drug selection and expanded for further experiments.

### Genomic PCR

Genomic DNA was extracted using ZR Genomic DNA II kit (Zymo Research Corp., Irvine, CA, USA) or QuickExtract™ DNA Extraction Solution (Epicentre Biotechnologies, Madison, WI, USA) according to the manufacturer’s instructions. Genomic PCR was performed with GoTaq® Green Master Mix (Promega Biosystems, Sunnyvale, CA, USA). Primers used are listed in Supplementary Table S1.

### Southern blotting

Genomic DNA was purified from cells by standard phenol/chloroform extraction and digested with ScaI-HF (New England Biolabs) for 6–8 h, supplemented with 1 mM spermidine (Sigma), 100 μg/ml bovine serum albumin (BSA; Sigma) and 50 μg/ml RNAse A (Millipore). About 10 μg digested DNA from each clone was separated on 0.8% agarose gels and transferred to hybond-N+ nylon membrane (GE Healthcare, Piscataway, NJ, USA). The GFP probe targeted to the GFP transgene was generated by PCR, and the primer sequences are listed in Supplementary Table S1. The labeling of probe and hybridization with the membranes were performed according to the manufacturer’s instructions (Amersham Rediprime II labeling system, GE Healthcare).

### Immunocytochemistry

Cells were fixed in 4% (wt./vol.) paraformaldehyde (Electron Microscopy Sciences, Hatfield, PA, USA) for 20 min, rinsed with phosphate buffered saline (PBS), permeabilized and blocked by 3% normal donkey serum (Jackson ImmunoResearch Laboratories, Bar Harbor, ME, USA) in PBST (PBS + 0.2% TritonX-100; Sigma) for 60 min at room temperature. Cells were incubated with primary antibodies in PBST at 4°C overnight and then washed with PBS three times. Then cells were incubated with secondary antibodies diluted in PBS containing 0.1% BSA (Jackson ImmunoResearch Laboratories) for 1 h at room temperature. Finally, the cells were counter-stained for nuclei with 1 μg/ml DAPI (Roche, Nutley, NJ, USA).

Primary antibodies used in this study include rabbit anti-OCT3/4, rabbit anti-SOX2, rabbit anti-NANOG, mouse anti-SSEA4 (all from Cell Signaling Technology, Beverly, MA, USA), mouse anti-TRA-1-60 (Millipore), mouse anti-TRA-1-81 (Millipore), mouse anti-AFP (Cell Signaling Technology), mouse anti-alpha-SMA (Abcam, Burlingame, CA, USA), mouse anti-βIII-Tubulin (Covance, Princeton, NJ, USA), goat anti-FOXA2 (R&D Systems), rabbit anti-OTX2 (Millipore) and rabbit anti-LMX1a (Millipore). Secondary antibodies were Alexa Fluor 488/594-conjugated donkey anti-mouse/rabbit/goat (Jackson ImmunoResearch Laboratories).

### Karyotyping

G-banded karyotyping was performed by WiCell Cytogenetic Services, Madison, WI, USA.

## RESULTS

### H11 *locus and DICE strategy*

We previously described a transcriptionally and recombinationally active, ubiquitously expressed murine locus called *Hipp11* (12–14). *Hipp11* is located in an intergenic region on chromosome 11, flanked by the two genes *Drg1* and *Eif4enif1* (12). The criteria for identification of the *Hipp11* locus included absence of disruption of regulatory elements or genes, as judged by sequence annotation, an intergenic region in a gene dense area, a location at the convergence between two genes transcribed in opposite directions and apparently ubiquitous transcriptional activity, as reflected by broad spatial and temporal expressed sequence tag expression patterns, indicating ubiquitous transcriptional activity. This latter feature is especially important in stem cells, where during differentiation, chromatin remodeling typically leads to silencing of some loci and potential activation of others. Within the region, a precise locus was chosen that was devoid of repetitive elements and conserved sequences and to which primers for amplification of homology arms could easily be designed. *In vivo* experiments verified that integration of targeting cassettes at *Hipp11* did not interfere with viability or fertility of mice and that biallelic expression of targeting cassettes was possible. Studies in mice confirmed that genes inserted at *Hipp11* displayed robust, ubiquitous expression that appeared to be superior to the expression levels of other commonly used ubiquitously active loci, including *ROSA26* (12–14).

To identify the orthologous locus in the human genome, we first located *DRG1* and *EIF4ENIF1* in human chromosomes and located the human equivalent locus, called *H11*, by its distance from these two genes. Human *DRG1* and *EIF4ENIF1* are located on chromosome 22q12.2, the distance between the two genes is very similar between mouse and human, and the intron/exon organization of *DRG1* and *EIF4ENIF1* is highly conserved between the two species. We screened the region ∼700 bp 3′ to the 3′ UTR of human *EIF4ENIF1* and chose a locus where primers could readily be designed for amplification of homology arms, 4500 bp downstream of *DRG1* and 683 bp downstream of *EIF4ENIF1* (Figure 1B). The level of DNA sequence identity between the mouse *Hipp11* and human *H11* regions was ∼45%, similar to the average sequence identity between mouse and human of 40%, reflecting the absence of highly conserved sequences in the region and consistent with a safe region for insertion of transgenes. Because of the parallels with the mouse location, we speculated that the *H11* locus would allow strong expression of inserted genes without disrupting endogenous gene function.

We then combined the human *H11* locus, spontaneous or TALEN-assisted HR and phiC31 and Bxb1 integrases to create an efficient site-specific integration system for ESC and iPSC. The overall strategy is shown in Figure 1A. The landing pad construct carried neomycin resistance and GFP genes for selection and screening, flanked by *attP* sites for phiC31 and Bxb1 integrases. This cassette was placed into the human *H11* locus by using either spontaneous or TALEN-assisted HR to generate recipient ESC and iPSC lines. Neomycin selection was used to identify clones that had integrated the landing pad, while GFP expression provided an indication of the level of gene expression from the locus.

In the second step, we performed DICE to introduce the genes of interest into the *H11* locus in the recipient cell lines. PhiC31 integrase and Bxb1 integrase were combined to generate a high degree of specificity and directionality for the cassette exchange reaction. The donor cassette carried puromycin resistance and mCherry genes for selection and screening, in addition to the gene of interest, for example, neural transcription factor genes. These donor genes were flanked by *attB* sites for phiC31 and Bxb1, to target site-specific recombination. PhiC31 and Bxb1 integrases recognize and recombine their *attP* and *attB* sites with a high degree of specificity using concerted cut and paste recombination events (18), resulting in all genes internal to the *attB* sites being inserted into the genome in a defined orientation and copy number. Therefore, in this system, DICE will ensue when the donor plasmid along with phiC31 and Bxb1 integrase-expressing plasmids are co-transfected into recipient cell lines. Through this two-step strategy, which combines genome editing methods mediated by HR and by site-specific integrases, we established a highly specific, precise and convenient system for editing ESC and iPSC genomes.

### Homologous recombination at the H11 locus and generation of recipient cell lines

**Figure 2.**
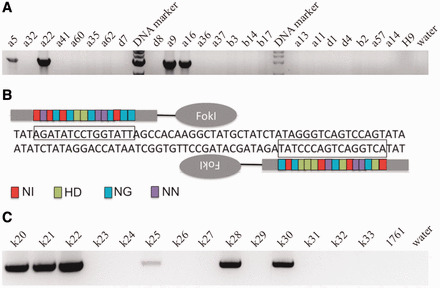
Generation of recipient ESC and iPSC lines. **(A)** Genomic PCR was used to screen for clones that underwent correctly targeted spontaneous HR in H9 ESC, producing integration of the landing pad cassette at the *H11* locus. A 3.6-kb band indicated correct targeting. **(B)** The TALEN pair used in this study to target the human *H11* locus. Color code for the amino acid positions 12 and 13 in a TALE repeat and the corresponding nucleotide in DNA: red NI for A, green HD for C, blue NG for T and purple NN for G. The double-strand break was generated ∼60 bp from the *H11* locus. **(C)** Genomic PCR was used to screen for clones that underwent correctly targeted placement of the landing pad by TALEN-assisted HR in PI-1761 iPSC. Representative clones are shown, with the expected 1.1 kb band indicating correct placement.

We first utilized spontaneous HR to insert the landing pad cassette into the *H11* locus in H9 ESC. The targeting vector contained an expression cassette carrying the neomycin resistance and GFP genes, flanked by the *attP* sites for phiC31 and Bxb1 integrases and two homology arms, of 5 and 3 kb (Supplementary Figure S1A). The targeting vector was linearized by digestion with SwaI and AscI and introduced into H9 cells by an optimized nucleofection protocol. G418 selection was started 2 days later and continued for 14 days, after which GFP-positive clones were picked and screened by genomic PCR and Southern blotting. Out of 98 clones analyzed, 6 underwent the desired recombination event (Figure 2A), indicating a targeting frequency of 6.1% in H9 ESC (Table 1). 

**Table 1.** Summary of HR experiments for generation of recipient ESC and iPSC lines

Cell line	Targeting method	Landing pad	Number of clones analyzed	Number of correctly targeted clones	Targeting efficiency (%)
H9 ESC	Spontaneous HR	5 kb arm-Neo-GFP-3 kb arm	98	6	6.1
1761 iPSC	Spontaneous HR	5 kb arm-Neo-GFP-3 kb arm	35	2	5.7
1754 iPSC	Spontaneous HR	5 kb arm-Neo-GFP-3 kb arm	23	0	0
H9 ESC	TALEN-assisted HR	0.4 kb arm-Neo-GFP-0.4 kb arm	12	7	58.3
1761 iPSC	TALEN-assisted HR	0.4 kb arm-Neo-GFP-0.4 kb arm	33	15	45.5
1754 iPSC	TALEN-assisted HR	0.4 kb arm-Neo-GFP-0.4 kb arm	24	13	54.2

The last column shows the percentage of correctly targeted clones for each condition.

We then asked whether iPSC could be genetically modified at the *H11* locus by the same method. For these experiments, we used PI-1754 iPSC from a PD patient (19) and PI-1761 iPSC from a normal sibling control. The PI-1754 iPSC line was generated from a PD patient with a triplication of the alpha-synuclein (*SNCA*) gene, and it exhibits PD-related pathological phenotypes in culture after differentiation into dopaminergic neurons (19). The landing pad vector for the iPSC was the same as that used for H9, except that the homology arm sequences were slightly different, reflecting minor DNA polymorphisms observed between these three human genomes (data not shown). Similar procedures were performed to carry out HR in PI-1754 and PI-1761 iPSC. For the normal sibling control PI-1761, two correctly targeted clones were identified out of 35 picked (5.7%; Table 1). However, we failed to get any correctly targeted clones among 23 clones picked for the PD patient-derived PI-1754 iPSC. This result may have been due to the disease-related phenotype of the PI-1754 iPSC, which is reflected in poor growth and propensity for differentiation upon passaging and after nucleofection. This phenotype is in contrast to the robust growth of the wild-type PI-1761 iPSC and H9 ESC lines, even after passaging and nucleofection.

In an attempt to stimulate the frequency of HR, which appeared critical for PI-1754 iPSC, we applied the recently described TALEN genome-editing method to generate double-strand breaks in a site-specific manner. Such breaks can be used to target the spontaneous HR system at an elevated frequency. TALEs are a newly described class of specific DNA-binding proteins from *Xanthomonas* plant pathogens (20). These proteins contain DNA-binding domains of TALE repeats composed of 33–35 amino acids. The TALE repeats have almost identical sequences, except for the ‘RVD’, which specifically recognize one target base. Repeats with the appropriate specificity are linked to a nuclease domain to generate a TALE-nuclease (TALEN). TALENs are straightforward to design and construct (10) and induce double-strand breaks in specific DNA sequences. The breaks will be repaired by the cell using either homology-directed repair or the error-prone process of NHEJ. TALENs have been shown to be effective for genome editing in human pluripotent stem cells (21), although as with other methods of artificially stimulating HR through provision of targeted double-strand breaks, off-target effects and unwanted mutagenesis can occur.

We identified two potential TALEN target sites (Spacer = 15–24 bp, RVDs = 15–20 bp) for the *H11* locus using the TAL Effector Nucleotide Targeter 2.0 (10,16), with one located exactly at the *H11* locus, while the other was 60 bp from the locus. TALEN pairs were assembled according to the Golden Gate methodology (10) (Figure 2B). We then determined the efficiency of these two pairs of TALENs to make double-strand breaks in the K562 cell line by a mutation detection kit. This assay is based on the generation of PCR products that are subsequently hybridized to generate mismatches in heteroduplexed DNA, and cleavage of the mismatches by a nuclease. According to this assay, the second TALEN pair (Figure 2B) showed a higher efficiency of double-strand breaks of ∼25% and was therefore chosen for genomic manipulation in ESC and iPSC (data not shown), even though it was farther from the locus.

For TALEN-assisted HR at the *H11* locus in ESC and iPSC, we used the same landing pad plasmid that was used for spontaneous recombination. However, shorter homology arms, of only 400 bp of homologous sequences flanking *H11*, were employed. We introduced the two TALEN expression vectors and the landing pad plasmid into H9 ESC and PI-1761 and PI-1754 iPSC by nucleofection. After similar neomycin drug selection and GFP screening procedures, genomic PCR analysis (Figure 2C), revealed that 7 of 12, 15 of 33 and 13 of 24 clones underwent the correct the recombination event in H9 ESC, and 1761 and 1754 iPSC, respectively, indicating a targeting efficiency of ∼40–50%, even for the PD patient 1754 iPSC (Table 1). These results represented a dramatic increase in recombination frequency of ∼8-fold at the *H11* locus in human ESC and iPSC mediated by TALEN-assisted HR. Therefore, the TALEN system was efficient for genomic editing, even in disease iPSC where spontaneous HR was ineffective.

### Characterization of ESC and iPSC recipient stem cell lines

**Figure 3.**
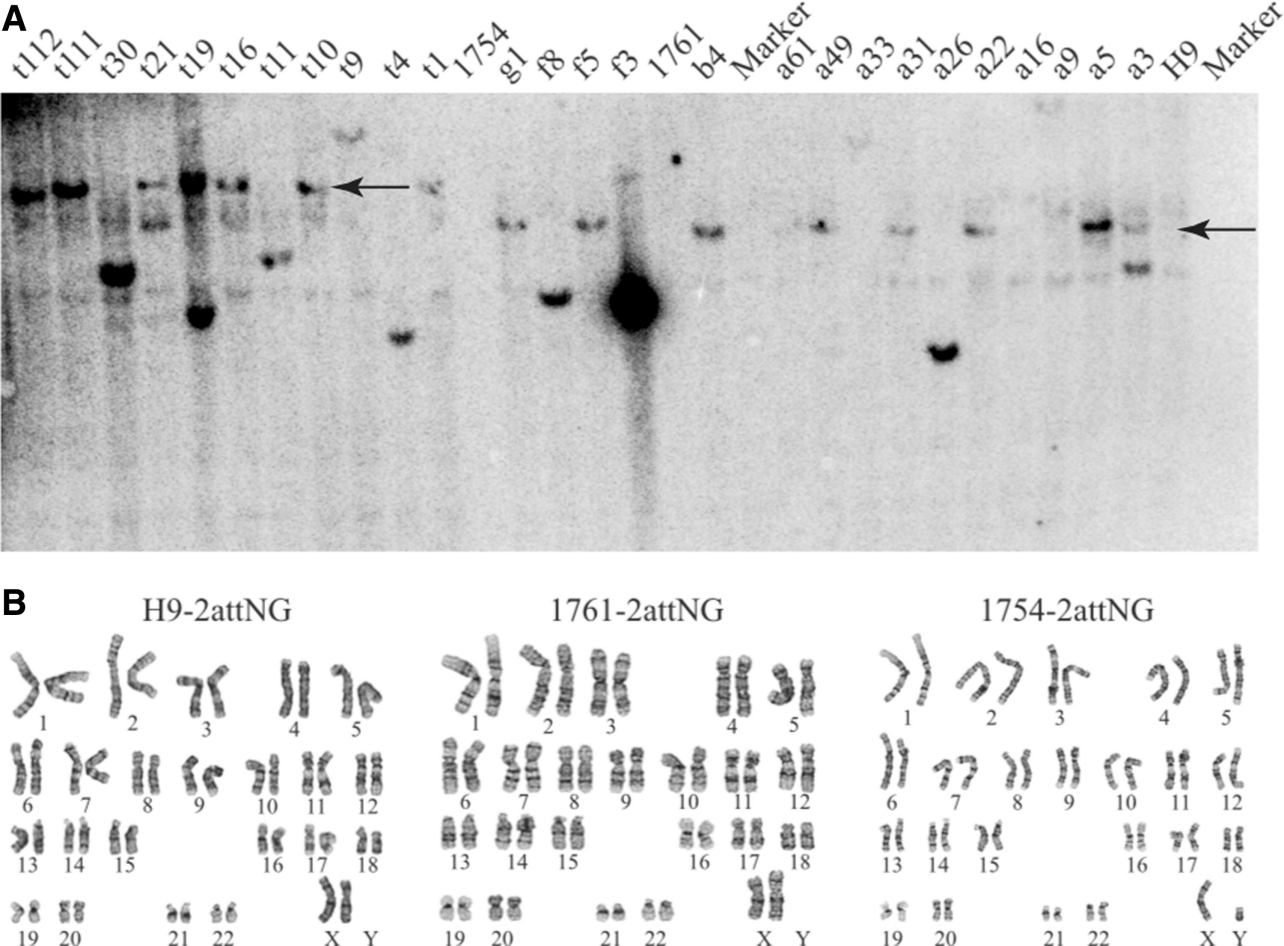
Southern blotting and G-banding analysis of recipient cell lines. **(A)** Southern blot analysis of selected clones. Marker lanes refer to size markers, with 9.1 kb for spontaneous HR and 12.0 kb for TALEN-assisted HR (arrows). Clones given the ‘a’ and ‘b’ prefixes were from H9 ESC targeted by spontaneous HR, while ‘f’ and ‘g’ prefixes indicate clones derived from PI-1761 iPSC, also targeted by spontaneous HR. The t clones were from PI-1754 iPSC targeted by TALEN-assisted HR. **(B)** G-banding analysis of recipient cell lines, including H9 recipient cell line H9-2attNG (a22), PI-1761 recipient cell line 1761-2attNG (f5) and PI-1754 recipient cell line 1754-2attNG (t17).

To identify ESC and iPSC recipient lines that have *attP* sites anchored at the *H11* locus for use in further experiments, we first screened some of the correctly targeted clones for integration copy number by Southern blotting, using a probe directed to the GFP gene in the landing pad cassette (Figure 1A). To determine whether one or both of the *H11* alleles had been recombined, we performed genomic PCR using primers targeting regions around *H11* in the human genome (Supplementary Figure S2B). The results showed that all clones tested that had been generated by spontaneous recombination had integrated a single copy of the landing pad, while some of the tested clones that resulted from TALEN-assisted recombination had integrated multiple copies (Figure 3A; Supplementary Figure S2A). Some of the single-copy clones were sequenced to examine whether there were any mutations in the region, including the landing pad cassette and the homology arms. Mutations were detected in regions around the double-strand break in some of the clones derived from TALEN-assisted recombination. These results suggest that residual TALENs may have created additional double-strand breaks after the desired recombination event occurred, and NHEJ may have mediated DNA repair, generating these mutations. Additional breaks could occur, because the TALENs were targeted to a region 60 bp from the H11 locus, so the intact target continued to exist, even after the correct recombination event occurred. From the clones that were single-copy and mutation free, we randomly selected one clone for each cell line, namely a22 for H9, f5 for PI-1761 and t17 for PI-1754, for further characterization as candidates to become validated recipient cell lines. Because we did not characterize all the clones, we do not know the frequency of mutation-free clones, but it was not difficult to find such clones. 

**Figure 4.**
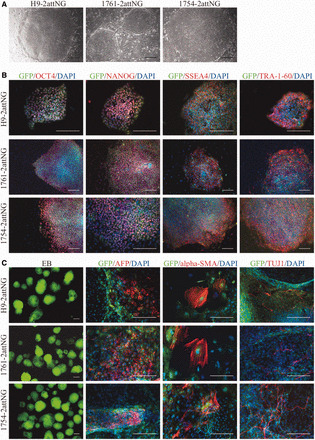
Pluripotency of recipient ESC and iPSC lines. **(A)** Typical ESC and iPSC morphology of the recipient cell lines H9-2attNG (left), 1761-2attNG (middle) and 1754-2attNG (right). **(B)** Immunostaining of pluripotent markers *OCT3/4*, *NANOG*, *SSEA4* and *TRA-1-60*, for H9-2attNG (upper), 1761-2attNG (middle) and 1754-2attNG (lower). **(C)** Typical morphology of suspended EBs at Day 8 (left) and immunostaining of marker genes for the three germ layers: AFP (endoderm), alpha-SMA (mesoderm) and Tuj1 (ectoderm) from H9-2attNG (upper), 1761-2attNG (middle) and 1754-2attNG (lower). Scale bar, 200 μm.

We tested whether these recipient lines, named H9-2attNG (a22), 1761-2attNG (f5) and 1754-2attNG (t17), remained pluripotent after genetic manipulation by immunostaining for pluripotency marker genes and by EB-mediated *in vitro* differentiation. All three cell lines exhibited the typical morphology of pluripotent ESC and iPSC (Figure 4A) and expressed GFP and a panel of pluripotency marker genes, including OCT3/4, SOX2, NANOG, SSEA4 and TRA-1-60 (Figure 4B). After 16 days of *in vitro* differentiation, all lines generated cells positive for markers of the three germ layers, including alpha-fetoprotein (endoderm), alpha-smooth muscle actin (mesoderm) and beta III-tubulin (ectoderm) (Figure 4C). Meanwhile, robust GFP expression was maintained in the three cell lines after extensive passaging (>30 passages) and after EB-mediated differentiation (Figure 4C), suggesting robust expression from the *H11* locus. Finally, G-banded karyotype analysis showed that all three recipient cell lines retained a normal karyotype (Figure 3B). 

In summary, recipient cell lines H9-2attNG ESC and 1761-2attNG and 1754-2attNG iPSC were successfully established, bearing phiC31 and Bxb1 integrase *attP* sites placed at the transcriptionally active *H11* locus. Because these *attP* sites are recognized and actionable by the phiC31 and Bxb1 integrases, the recipient cell lines can be used to insert and express any genes of interest that are flanked by phiC31 and Bxb1 *attB* sites.

### Dual integrase cassette exchange in H9-2attNG

**Figure 5.**
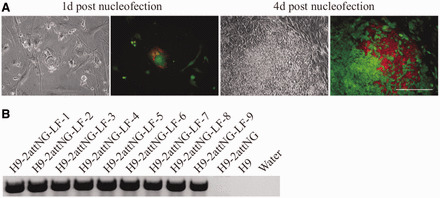
DICE reaction mediated by phiC31 and Bxb1 integrases. **(A)** The visible change in marker gene expression as mCherry replaces GFP 1 and 4 days after nucleofection. These cells may not be a single clone, as ESC tends to grow as clusters. Puromycin drug selection is used to obtain pure recombinant cells. It takes ∼2 days for the cells to completely convert to mCherry expression. **(B)** An example of genomic PCR for screening of correctly targeted clones after DICE, yielding a band of 1.2 kb. H9-LF (LMX1a-FOXA2) clones are shown. Scale bar, 200 μm.

A key feature of our strategy is that transient expression of phiC31 and Bxb1 integrases in recipient cell lines will catalyze site-specific, irreversible recombination between cognate *attP* and *attB* sites, resulting in precise replacement of the landing pad cassette by any donor cassette flanked by phiC31 and Bxb1 *attB* sites. We named this process DICE (Figure 1A). To determine the efficiency of DICE in a recipient cell line, we constructed a control donor vector in which phiC31 and Bxb1 *attB* sites flanked genes for the puromycin resistance selection marker and the mCherry fluorescent marker protein, driven by the PGK promoter (Supplementary Figure S1B). We introduced this vector together with phiC31 and Bxb1 integrase expression vectors into the H9-2attNG recipient cell line by nucleofection, and started puromycin drug selection 3–5 days later, for 2–3 days. Within several days, the change in marker gene expression from GFP to mCherry was visible (Figure 5A). We did not observe any puromycin-resistant clones that were mCherry-negative, and all the puromycin-resistant clones showed complete conversion of cells from green to red. This result was expected, as puromycin and mCherry are expressed by the same promoter, linked by P2A. After 2 weeks, 12 puro-resistant clones that were mCherry-positive and GFP-negative were picked at random. We tested whether DICE had occurred in these clones by performing genomic PCR using primers designed to detect the landing pad or donor cassettes. The results showed that all 12 of the clones (H9-PC) carried the puromycin–mCherry donor cassette at the *H11* locus, while the landing pad cassette was no longer present. This result is consistent with DICE having successfully occurred in 100% of the picked clones (Figure 5B; Supplementary Figure S3). 

### Overexpression of neural transcription factors in recipient stem cell lines

We further asked whether the DICE genomic editing system could be employed as a rapid method to create cell lines that may be useful to optimize differentiation protocols into specific lineages by overexpressing relevant transcription factors. The transcription factors *Lmx1a*, *FoxA2* and *Otx2* were previously shown to be critical for dopaminergic neuron development in mouse (22). Forced expression of these factors individually or in combinations in ESC improved neuronal differentiation (23–25). We hypothesized that overexpression of these factors specifically at the neural stem cell stage during directed differentiation from ESC or iPSC might be effective in increasing differentiation efficiency into dopaminergic neurons. To this end, we designed a series of four constructs, based on the control p2attPC donor vector, that carried the human *LMX1a* (*L*), *FOXA2* (*F*) and *OTX2* (*O*) coding sequences in all pairwise combinations and with all three together. GFP (*G*) was also included as a reporter, and the entire cassette was driven by the NESTIN enhancer and minimal thymidine kinase promoter. With this design, we expected that, at the stage of neuronal differentiation when the neural progenitor marker NESTIN is expressed, the inserted genes would be expressed as well.

We used ribosomal skipping 2A sequences, including 2A peptide regions from equine rhinitis A virus (‘E2A’), porcine teschovirus-1 (‘P2A’) and insect *Thosea asigna* virus (‘T2A’), to generate multicistronic vectors for efficient translation of the inserted genes (26,27) (Supplementary Figure S1B). 2A peptides have been reported to mediate stoichiometric production of separate proteins from dicistronic or multicistronic genes in multiple cell types (26). The sizes of the transgene cassettes present between the two *attB* sites ranged from ∼5 to 7 kb. We introduced phiC31 and Bxb1 integrase expression plasmids and each of the four different constructs into H9-2attNG and carried out similar nucleofection, selection and analysis procedures to those described above. Genomic PCR analysis indicated that all clones (named H9-LFO, H9-LF, H9-LO and H9-OF) tested had the desired donor cassette present at the *H11* locus, whereas the original NG landing pad was no longer present, again indicating that DICE is 100% specific (Figure 5B; Table 2). 

**Table 2.** Summary of DICE results in recipient ESC and iPSC lines

	PC positive clones/all clones	LFO positive clones/all clones	LO positive clones/all clones	LF positive clones/all clones	FO positive clones/all clones
H9-2attNG	12/12	12/12	12/12	9/9	7/7
1761-2attNG	17/17	11/11	7/7	3/3	5/5
1754-2attNG	5/5	4/4	2/2	3/3	2/2

PC, puromycin-mCherry; L, LMX1a; F, FOXA2; O, OTX2. Correct addition of marker genes alone or marker and transcription factor genes occurred in 111/111 of the mCherry-positive, GFP-negative clones screened.

To determine whether DICE would also be effective in iPSC, we introduced the control p2attPC plasmid and each of the four transcription factor constructs into the 1761-2attNG and 1754-2attNG recipient iPSC lines. After co-transfection of phiC31 and Bxb1 integrase expression plasmids and the donor construct and puromycine selection, GFP-negative and mCherry-positive clones were picked randomly. Genomic PCR was used to detect which clones had undergone cassette exchange. Although fewer puromycin-resistant colonies were obtained from patient 1754 iPSC than from the normal sibling control 1761 iPSC, all the randomly chosen clones were positive for the desired cassette exchange, as summarized in Table 2.

We then randomly chose two clones for further experiments representing H9-LFO, carrying LMX1a, FOXA2, OTX2 and GFP, and H9-PC, carrying only the puromycin and mCherry genes and no transcription factors. Both clones expressed a panel of typical pluripotentcy markers, including OCT3/4, NANOG, SSEA4 and TRA-1-60 (Supplementary Figure S4). Both clones also produced cells representative of the three germ layers after EB-mediated differentiation (Supplementary Figure S5). To test further whether the pluripotency of the lines was affected by the DICE procedure, H9 clones carrying various transcription factors that had been added by DICE were compared to H9 cells in which transcription factors were not added. End-point reverse-transcription PCR was carried out for five pluripotency genes in the engineered and control H9 cells (Supplementary Figure S6). Band intensity of pluripotency genes was comparable in all the lines.

**Figure 6.**
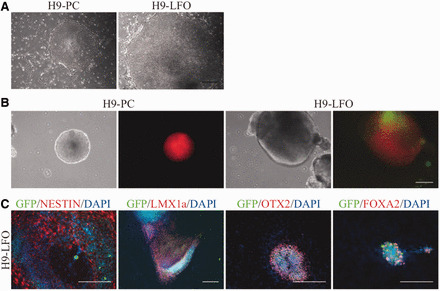
Characterization of overexpression of neural transcription factors in correctly targeted clones. **(A)** Typical ESC morphology of H9 clones after DICE. H9-PC (control, left) and H9-LFO (LMX1a-FOXA2-OTX2, right) are shown. **(B)** The morphology of suspended EBs and the expression of mCherry and GFP at Day 8 from H9-PC (left) and H9-LFO (right). **(C)** Immunostaining of NESTIN, LMX1a, OTX2 and FOXA2 in the H9-LFO line after 16 days of EB-mediated differentiation. Endogenous mCherry expression was weak and not detected by the fluorescence microscope used, so did not interfere with target protein staining. Scale bar, 200 μm.

To determine whether the three transcription factors were overexpressed in a neuronal cell-specific manner in H9-LFO, we examined expression of the neuronal progenitor marker NESTIN in EB-differentiated cells by immunostaining and GFP by fluorescence (Figure 6). NESTIN was turned on in some cells during EB-mediated differentiation, and these cells were also labeled by GFP expression, consistent with NESTIN-regulated expression of the transcription factor cassette. In contrast, no GFP-positive cells were detected in EB-differentiated cells from the control H9-PC (Figure 6B). 

Immunostaining was then performed to examine the expression of the three transcription factors at Day 16 of differentiation. The results showed that in differentiated cells from the H9-PC control line, only a few cells expressed FOXA2 and almost no cells expressed LMX1a or OTX2, reflecting the expected low endogenous expression pattern of these three transcription factors in randomly differentiated cells. However, in cells differentiated from the H9-LFO line, there were more cells expressing LMX1a, FOXA2 or OTX2, and most of these cells were also positive for GFP, suggesting that the expression of these introduced genes was activated by the exogenous NESTIN enhancer (Figure 6C). Together these results suggested that ESC and iPSC genomes could be efficiently modified by the DICE method and that genes of interest could be faithfully expressed at the *H11* locus, even after differentiation.

## DISCUSSION

In this study, we developed a novel strategy to achieve stringent site-specific integration in human ESC and iPSC by combining TALEN-assisted HR at a newly described locus for robust expression with DICE for rapid and precise gene insertion. In our system, we first established recipient H9 ESC and PD and sibling control iPSC lines that carried a landing pad with *attP* sites for phiC31 and Bxb1 integrases. This step was accomplished by either spontaneous or TALEN-assisted HR. The correct recombination event occurred at a frequency of ∼6% by spontaneous recombination and 40–50% by TALEN-assisted recombination, illustrating the benefit of TALENs to stimulate recombination. In the case of the PD line, recombination was only successful when TALENs were used. We then induced DICE by introducing plasmids encoding phiC31 and Bxb1 integrases to mediate site-specific integration at the landing pad. We co-introduced genes for mCherry and/or the dopaminergic neuron-inducing transcription factors LMX1a, OTX2 and FOXA2 in the context of a puromycin resistance selection marker cassette into the recipient cell lines, achieving successful cassette exchange with a specificity of 100% after screening and selection. We demonstrated that transgenes inserted into the *H11* locus were faithfully expressed in long-term culture of ESC and iPSC and were expressed in cells of all three germ layers after differentiation. Therefore, this genomic editing system provides a methodology that will enable the use of human ESC and iPSC in studies exploring gene function, lineage tracking, disease modeling, drug discovery and other goals.

Here, we have identified and targeted, for the first time, the *H11* human homolog of the mouse *Hipp11* locus (12). *H11* is in a transcriptionally active intergenic region of human chromosome 22 that appears to be highly suitable as a safe and effective location for placement of transgenes in human cells. Our present study showed that the targeting efficiency at *H11* was high—approximately 5–6% by spontaneous recombination and 40–50% by TALEN-assisted HR, both of which are higher than typically reported frequencies and suggestive of open chromatin. In addition, transgenes placed at the *H11* locus were actively and faithfully expressed without apparent silencing for over 30 passages. We mapped 300 kb of DNA sequence flanking the *H11* locus in the human genome utilizing the UCSC database and found that there were no cancer genes present in the region and only one microRNA, 288 kb distant from *H11*. In these respects, *H11* compares favorably with the current widely used loci for transgene insertion in human cells, *ROSA26*, *AAVS1* and *HPRT1*, all of which are in exons or introns of endogenous genes and are differentially expressed in different tissues (28). Therefore, *H11* appears to be an excellent locus for a wide variety of genome editing purposes.

In addition, for the first time, we employed two different integrases simultaneously, phiC31 and Bxb1, to create a more effective cassette exchange strategy. This strategy conferred significant advantages, as the use of dual integrases provided high specificity and efficiency. Previous cassette exchange studies utilizing serine integrases have employed phiC31 integrase alone, in *Schizosaccharomyces pombe* (29), *Drosophila* (30) and mouse cells and embryos (1,2,13,31). In these cases, unwanted side reactions occurred in which plasmid integration rather than cassette exchange occurred, or cassette exchange occurred in the wrong orientation, making it necessary to screen for the desired reaction. By using two different integrases with mutually exclusive *att* site recognition, these problems were effectively eliminated. We found in this study that 111/111 cassette exchange reactions analyzed had undergone the desired recombination event (Table 2). In one study (4), a single phiC31 *attP* site was randomly integrated and successfully used to target integration of an *attB* donor plasmid. However, the use of random integration to place the landing pad reduced positional control, and the use of a single integrase site rather than cassette exchange necessitated the use of a second, FLP-mediated, step to remove plasmid sequences (4).

The hypothesis that the combined use of two highly site-specific integrases would allow more controlled and specific cassette exchange appears to have been validated by this study. It is also notable that no limitation on the size of the incoming DNA sequence is expected with this system. We tested different constructs carrying transgene sizes between the two *attB* sites ranging from 3 to 7 kb, and all the sequences were successfully inserted into the desired locus in our recipient ESC and iPSC lines. In principle, it should be possible to insert much larger gene cassettes with this system, as has been observed with phiC31 integrase alone (5), and this feature is currently being tested.

In human and other mammalian cells, phiC31 integrase is known to be capable of recombining with certain native sequences called pseudo *attP* sites that bear partial identity to its wild-type *attP* site (32–35). This behavior is undesirable for DICE, because it could contribute to a background of undesirable recombination events. To minimize the possibility of pseudo site integration in our study, we employed a phiC31 expression vector, pCS-kI (17), that appears to diminish pseudo site integration without impacting wild-type *attP–attB* recombination (data not shown). This kind of phiC31 activity without pseudo site interference has also been observed by others (1–4,^31^). Work is presently underway in our laboratory to understand the parameters that are necessary to obtain these different behaviors from phiC31 integrase, and we plan to address this subject in a future study.

Genome editing technology is rapidly advancing, and during our preparation of the H9 ESC recipient cell line by classical spontaneous HR, the TALEN method for stimulating recombination efficiency was developed (10,20,21). Although we confirmed that TALEN-assisted HR occurred at an 8- to 10-fold higher frequency than spontaneous recombination (Table 1), this new method also brought some problems that necessitated careful screening of recombinants. First, it increased the occurrence of multiple-copy integration (Figure 3), whereas all clones derived by spontaneous recombination were single-copy. Mutations in the vicinity of the double-strand break were also observed in some TALEN-derived clones. Given these issues, once a validated, single-copy, mutation-free recipient cell line is generated by TALEN-assisted recombination, it may be faster and safer to carry out multiple rounds of gene addition by using highly specific integrases, rather than relying upon repeated exposure of the cells to TALENs, which may produce undesirable recombinational and mutagenic effects through exposure of cells to double-strand breaks. In contrast, integrase-mediated exchange at wild-type *att* sites is a concerted strand-exchange reaction that does not produce free double-strand breaks (18) and has been found to be invariably precise to the base (2,6,31,36) (C. Zhao *et al.*, submitted for publication).

To compare available methods for genome modification in human pluripotent stem cells, methods relying on random integration have the serious limitation of lack of control of the genomic target site, which leads to position effects on transgene expression and potential perturbation of endogenous gene expression. Use of HR to control target site relieves these problems. Endogenous HR is safe, but relatively inefficient. Recombination stimulated by TALEN or CRISPR/Cas systems increases recombination efficiency, although off-target effects and introduction of mutations near the target site are potential drawbacks. The DICE system is initially more laborious than use of a TALEN or CRISPR/Cas system alone, as DICE requires two steps. However, once the landing pad is inserted, the DICE system allows for quick and precise genomic editing with a higher efficiency and a clean background. Therefore, particularly when one desires making multiple lines with different insertions in the same location, the DICE system is highly advantageous.

In summary, the combination of HR with DICE mediated by phiC31 and Bxb1 integrases represents a practical strategy for rapid and stringent genome editing in human pluripotent stem cells. After creating a recipient cell line harboring phiC31 and Bxb1 *attP* sites, subclones with confirmed insertion of any gene of interest can easily be obtained in as short as 2–3 weeks. Positioning transgenes at the safe, intergenic, transcriptionally active *H11* locus allows diverse genetic manipulations, including comparison of the functions of different genes in the same background and during differentiation. While demonstrated here in human ESC and iPSC at the *H11* locus, the DICE system is likely to be successful in a wide variety of additional cell types, species and genomic contexts.

## FUNDING

California Institute for Regenerative Medicine [RT2-01880 and TR2-01756]. Funding for open access charge: California Institute for Regenerative Medicine [RT2-01880 and TR2-01756].

*Conflict of interest statement*. M.P.C. is an inventor on Stanford-owned patents covering phiC31 integrase.

## Supplementary Material

supplementary_data
