# Supplementary material for: DICE, an efficient system for iterative genomic editing in human pluripotent stem cells
Source: Nucleic Acids Res. 2013 Dec 4;42(5):e34. doi: 10.1093/nar/gkt1290 (PMC3950688; doi:10.1093/nar/gkt1290)
Supplement: supplementary_data [file supplementary_data.zip › supp_gkt1290_nar-02543-met-h-2013-File008.pdf]

SUPPLEMENTARY MATERIAL

Name	Sequence (5' to 3')	Purpose
302	ATCGGCGGCCGCGGCGCGCCCCTTTTTCCTTGAGCTTTAAA GACCCCAACAGGTCAG	5' HR homology arm F
303	ATCGGTCGACGCTCTTGGGAAGAAGTCAAACATTATTTCAG	5' HR homology arm R
301	ATCGGTCGACGGCGCGCCATTTAAATGTTTAAACCCATAGTTG ATTTCTCCTAAATCAAGATAGAGTCC	3' HR homology arm F
305	ATCGGCGGCCGCCCGCGGTTAATTAACCAAATGATTAATCCT GATGGCTGAGGAGAC	3' HR homology arm R
H11 5-1	AATTATTTAAATGACTCAGAACTGTACTGTAT	5' TALEN homology arm F
H115-2	AATTAGATCTCCTTTTTCCTTGAGCTTTAA	5' TALEN homology arm R
H11 3-1	AATTCCATAGTTGATTTCTCCTAA	3' TALEN homology arm F
H11 3-2	AATTGGCGCGCCAGTATGTTCCTTTAAGGAAG	3' TALEN homology arm R
T9	ACCCAGTCCGCCCTGAGCAA	HR master gPCR F
HN3R1	TGGGCAGAAGGGAGGGTATGGACATGTAAG	HR master gPCR R
H4005F4	AGTTCCAGGCTTATAGTCATTATTCCCTAA	TALEN master gPCR F
N-5R3	GTCTCATGAGCGGATACATATTTGAATGTA	TALEN master gPCR R
Gprobe F	GACGGCGACGTAAACGGCCA	Southern G probe F
GprobeR	TTGCTCAGGGCGGACTGGGT	Southern G probe R
H400-5F1	CCAACCACCTTGACCTTTACCTCATTATCT	Second allele F/HR DICE R
N2	CTGCAGTCCAGCCTGGCGAC	Second allele R
CE-bxb1-F2	ACGGCGGTCTCCGTCGTCAG	HR DICE F
H400-5F2	AAGCTGAGGAATCACATGGAGTGAATAGCA	TALEN DICE F
CE-attB-R2	GGGTGGGGCAGGACAGCAAG	TALEN DICE R

Supplementary Table 1. List of key primers used in this study.

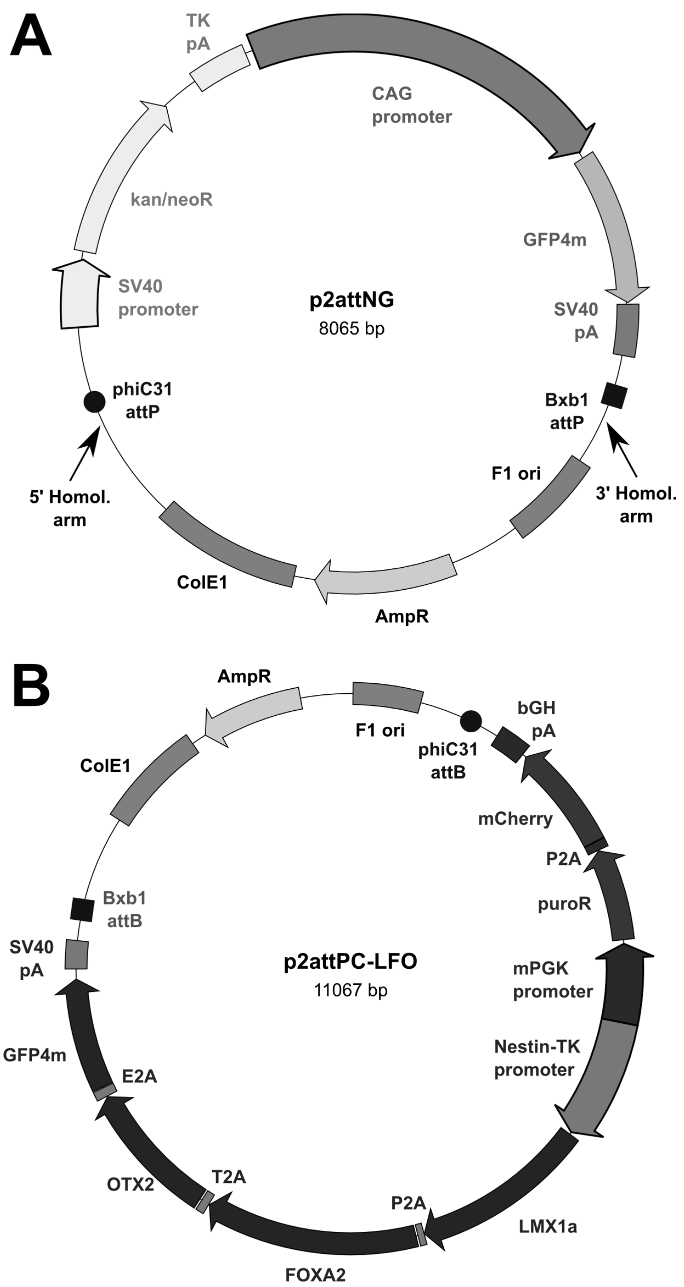

Supplementary Figure 1. Plasmid maps of the landing pad construct (A) and the donor cassette construct (B), where the LFO plasmid bearing three transcription factors (LMX1a-FOXA2-OTX2) is shown as an example. The phiC31 and Bxb1 *attP* site fragments used in p2attNG were 221 bp and 253 bp in length, respectively. In all donor cassette constructs, the phiC31 and Bxb1 *attB* site fragments were 285 bp and 300 bp in size, respectively. Homology arms (not shown) were added to p2attNG upstream of the phiC31 *attP* site and downstream of the Bxb1 *attP* sequence (arrows indicate positions where arms were cloned in p2attNG derivatives). The GFP4m gene is a variant of EGFP (mut4EGFP) that exhibits improved folding at 37°C (38). E2A, equine rhinitis A virus 2A peptide; P2A, porcine teschovirus-1 2A peptide; T2A, *Thosea asigna* virus 2A peptide.

A

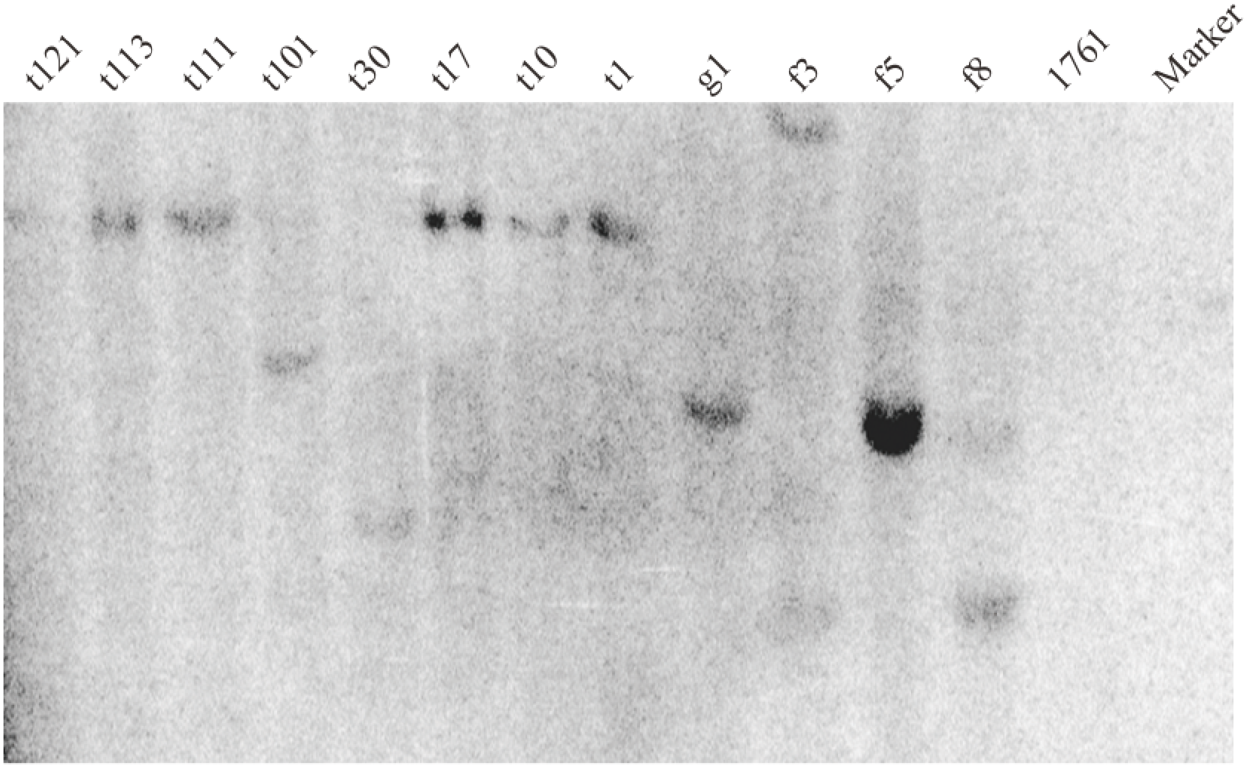

B

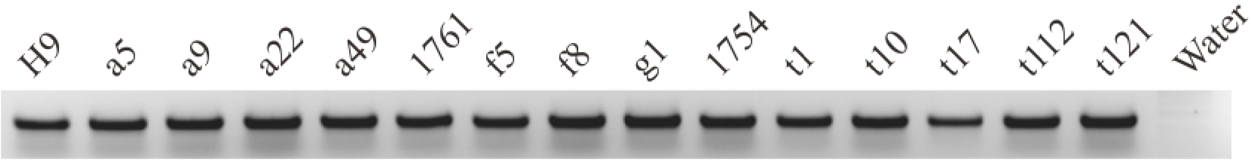

Supplementary Figure 2. Assays for copy numbers of transgenes in selected clones. (A) Southern blotting analysis for selected recipient cell lines. (B) Genomic PCR analysis for the second allele of the *H11* locus showed that only one allele is targeted in all cell lines tested. “a” and “b” clones come from H9 targeted by spontaneous homologous recombination; “f” and “g” come from PI-1761 targeted by spontaneous homologous recombination; “t” clones come from PI-1754 targeted by TALEN-assisted homologous recombination.

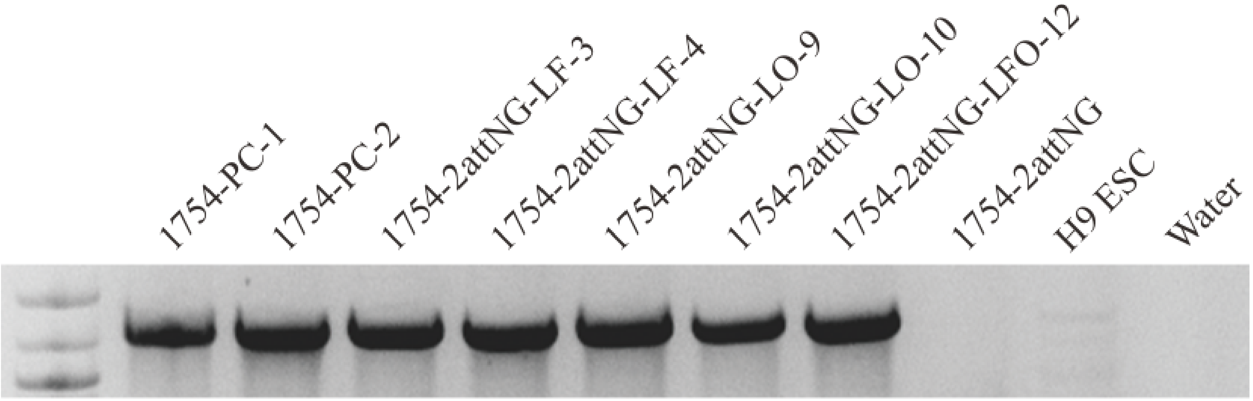

Supplementary Figure 3. DICE analysis. An example of the genomic PCR carried out to screen for correctly targeted clones after DICE. Clones derived from 1754-2attNG are shown on this gel.

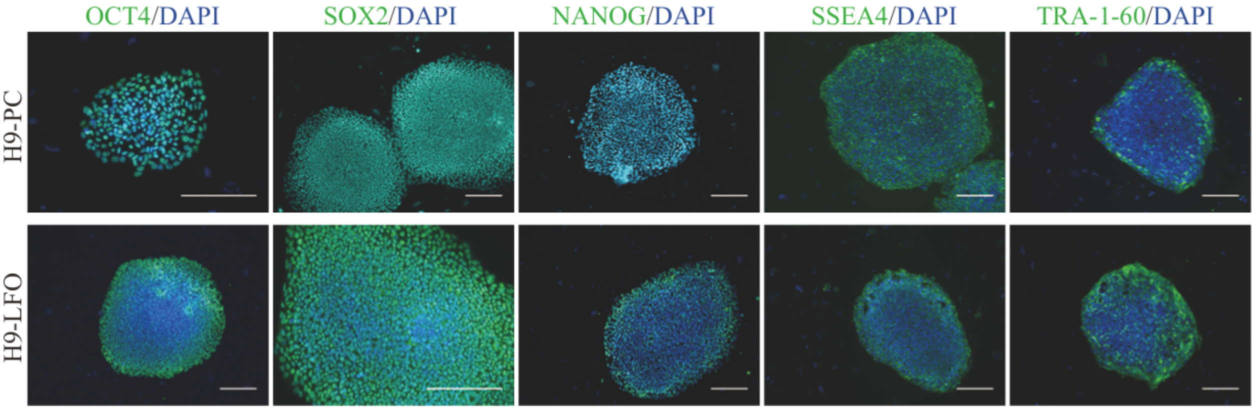

Supplementary Figure 4. Pluripotency analysis. Immunostaining of pluripotentcy markers OCT3/4, SOX2, NANOG, SSEA4, and TRA-1-60, in H9-derived lines after DICE. H9-PC (upper) and H9-LFO (lower) are shown. Scale bar, 200 μm.

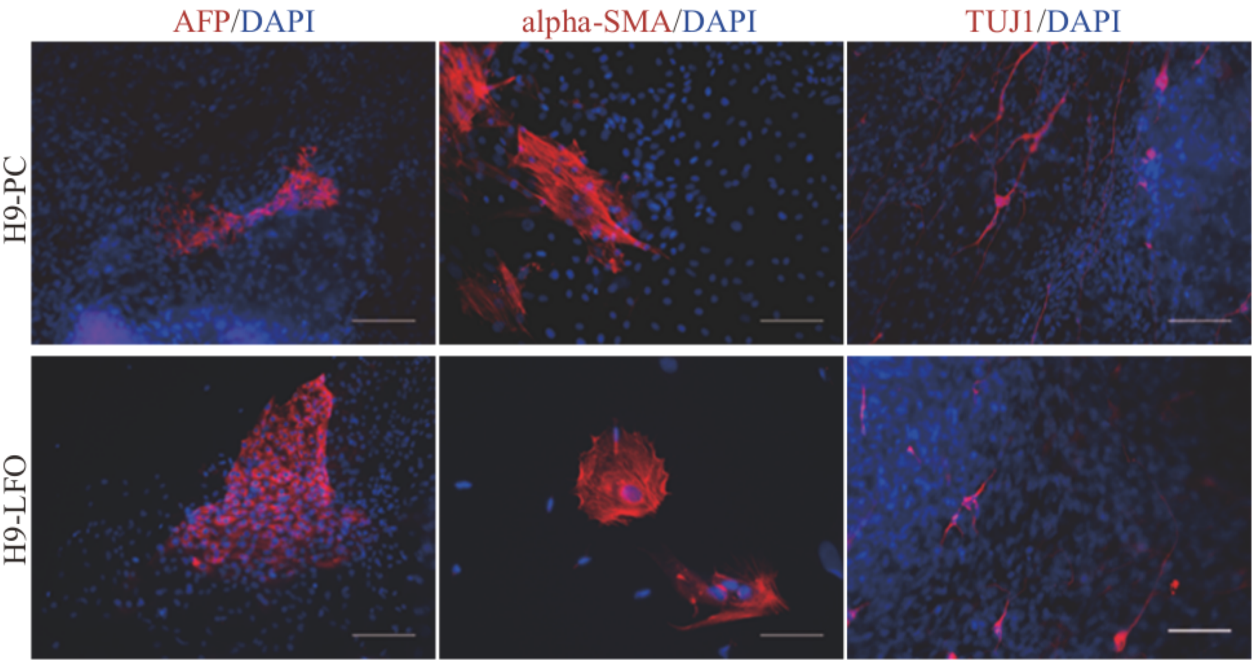

Supplementary Figure 5. Immunostaining of markers for the three germ layers in H9-derived lines after DICE: AFP (endoderm), alpha-SMA (mesoderm), and Tuj1 (ectoderm). H9-PC (upper) and H9-LFO (lower) are shown. Scale bar, 200 μm.

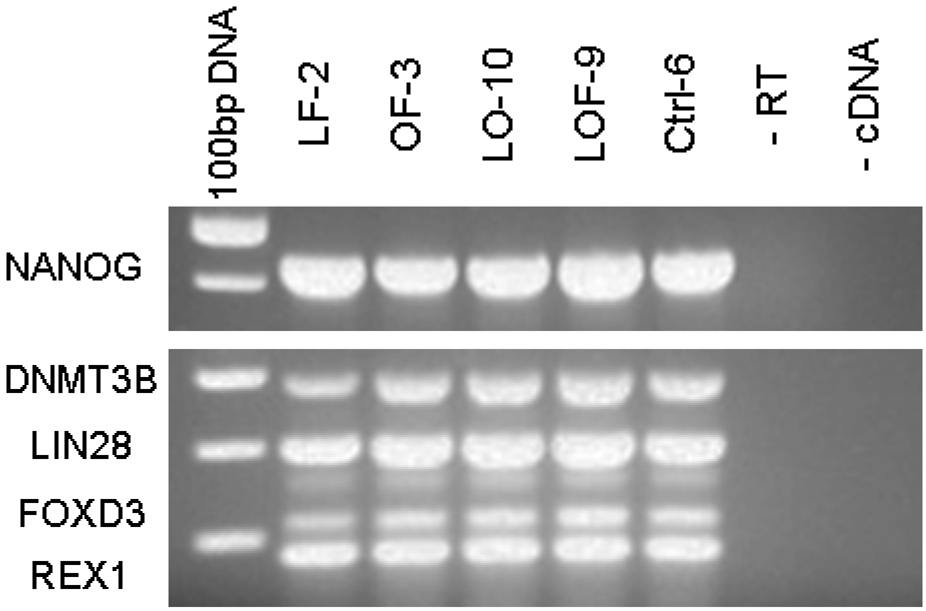

Supplementary Figure 6. End-point reverse-transcription PCR (RT-PCR) for five pluripotency genes assayed in engineered H9 ESC lines. Multiplex RT-PCR shows products for NANOG (438-bp), DNMT3B (380-bp), LIN28 (300-bp), FOXD3 (218-bp), REX1 (180-bp) at comparable intensities for all five cell lines.

NEB 100-bp DNA marker (NEB, Cat. No. N3231S), negative controls: -RT without reverse transcriptase, -cDNA without cDNA template.

SUPPLEMENTARY METHODS

End-point reverse-transcription PCR

iPSC colonies were collected by scraping the cells and collecting them by centrifugation. Total RNA was extracted using RNeasy Micro Kit (Qiagen, Cat.No. 74004) according to manufacturer's instructions. For the first strand cDNA synthesis, 200 ng total RNA was reverse transcribed with iScript cDNA Synthesis Kit (Biorad, Cat. No. 170-8891). We designed a multiplex PCR for five pluripotency markers, REX1, 180 bp; FOXD3, 218 bp; LIN28, 300 bp; DNMT3B, 380 bp, and NANOG, 438 bp. Primer sequences are available upon request. For the PCR, we used Top Taq Master Mix Kit (Qiagen Cat. No. 200403) with 56ºC annealing temperature and 35 cycles. PCR products were separated on 2% agarose gel.

SUPPLEMENTARY REFERENCES

38. Okada, A., Lansford, R., Weimann, J. M., Fraser, S. E., and McConnell, S. K. (1999). Imaging Cells in the Developing Nervous System with Retrovirus Expressing Modified Green Fluorescent Protein. *Exp Neurol.*, **156**, 394-406.
