# Supplementary figures and images for: DICE, an efficient system for iterative genomic editing in human pluripotent stem cells
Source: Nucleic Acids Res. 2013 Dec 4;42(5):e34. doi: 10.1093/nar/gkt1290 (PMC3950688; doi:10.1093/nar/gkt1290)

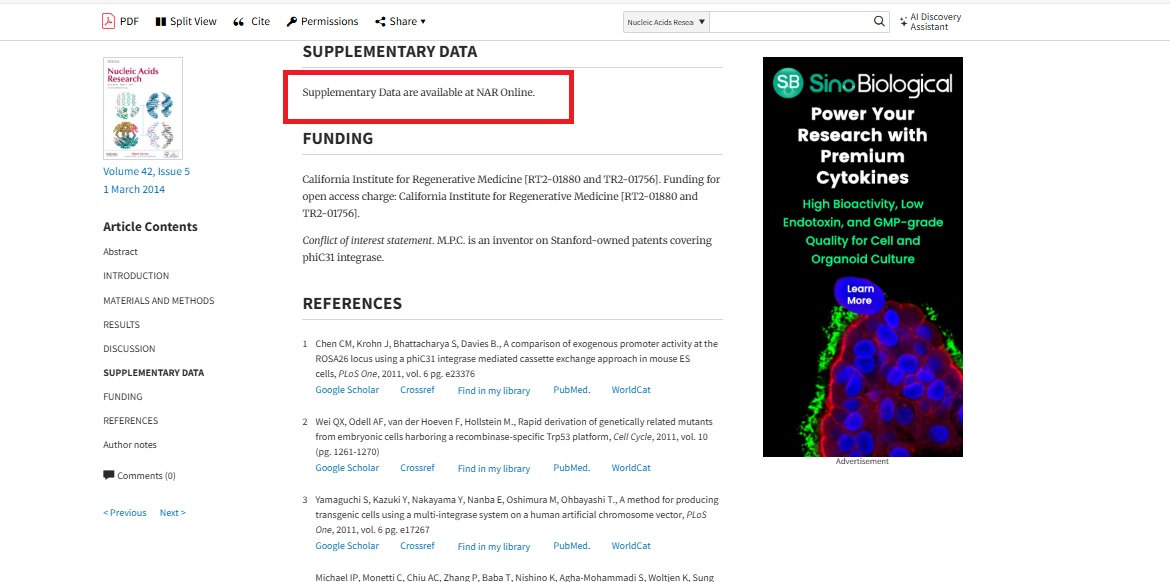

Supplement: supplementary_data [file supplementary_data.zip › Supplementary data area.jpg]
